# Supplementary material for: A Sesquiterpene Synthase from the Endophytic Fungus Serendipita indica Catalyzes Formation of Viridiflorol
Source: Biomolecules. 2021 Jun 16;11(6):898. doi: 10.3390/biom11060898 (PMC8234273; doi:10.3390/biom11060898)
Supplement: Supplementary file 1 [file biomolecules-11-00898-s001.zip › biomolecules-1248705 supplementary.pdf]

A sesquiterpene synthase from the endophytic fungus *Serendipita indica* catalyses formation of viridiflorol

Fani Ntana [1], Wajid W. Bhat [2], Sean R. Johnson [3], Hans J. L. Jørgensen [4], David B. Collinge [4], Birgit Jensen [4] and Björn Hamberger [2,*]

SUPPLEMENTARY FIGURES

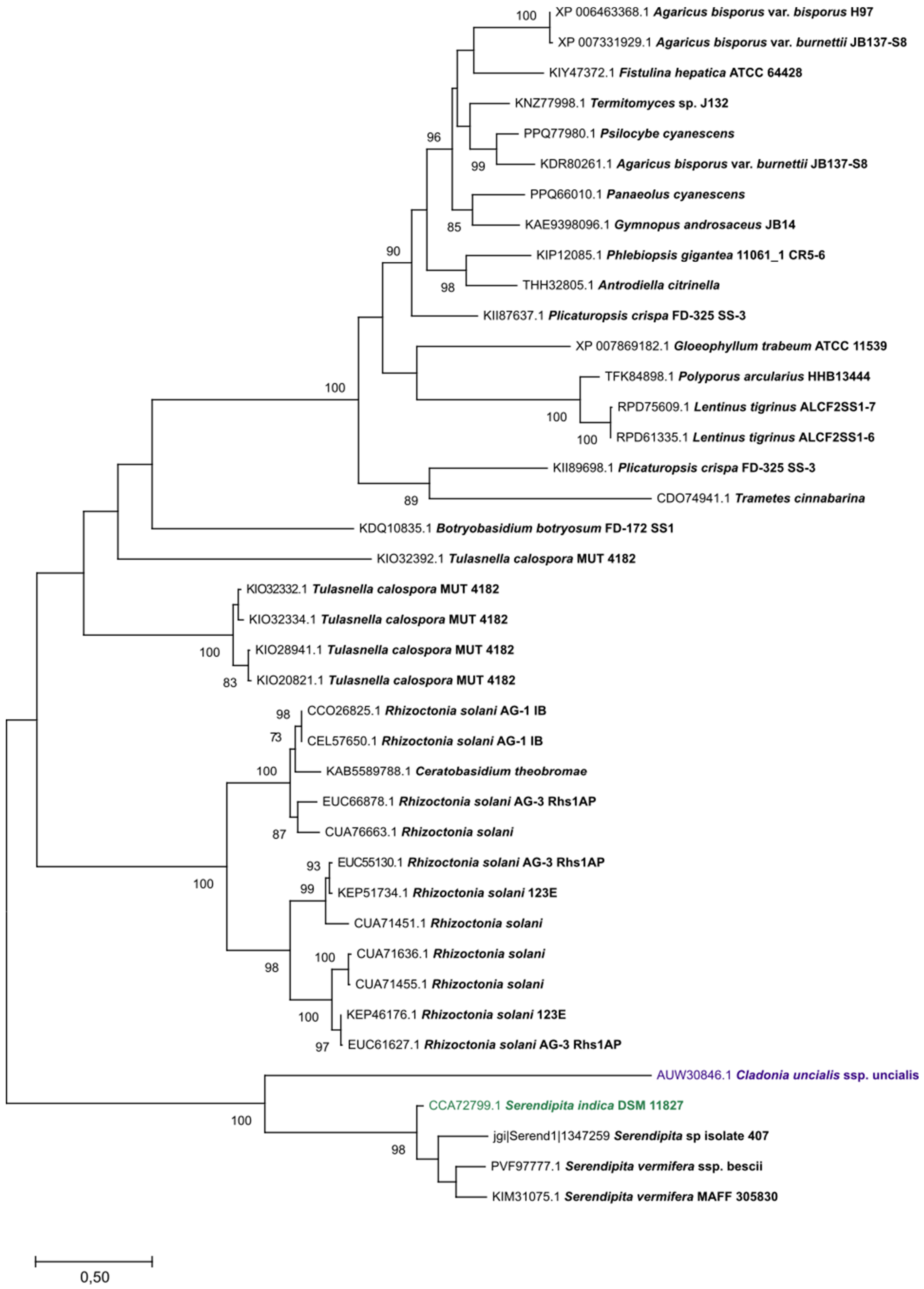

Figure S1. Phylogenetic analysis of SiTPS and related sequences. A Maximum-likelihood tree was constructed (bootstrap of 500), using a selection of proteins (NCBI NR, threshold of E-value 2e-67) similar

to SiTPS (shown here in green) and all the putative TPSs from other *Serendipita* species. The TPS from *Cladonia unciallis* (AUW30846.1) is marked in purple. The scale bar indicates a genetic distance of 0.50 and the bootstrap values are shown below the branches.

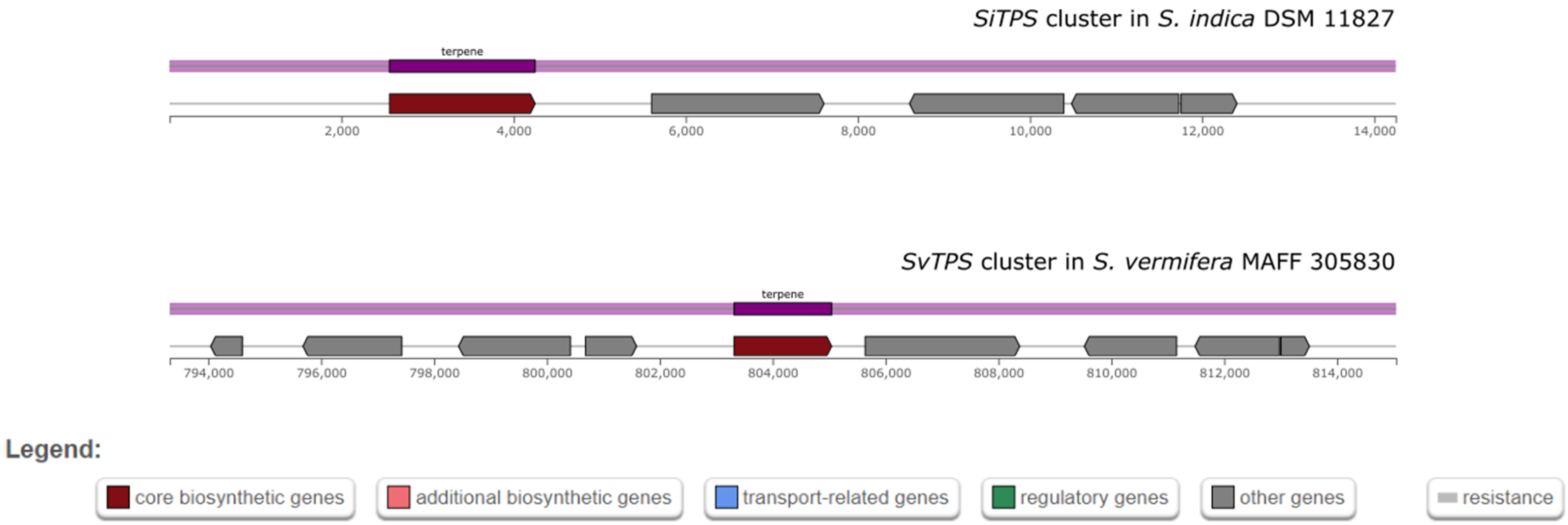

Figure S2. Terpene biosynthetic clusters of *SiTPS* and *SvTPS* in *S. indica* and *S. vermifera*, respectively. No other relevant to terpenoid biosynthesis or transport-related gene is located close to the two *TPS* genes. Results displayed have been generated after analysis of the two fungal genomes with the antiSMASH fungal version.

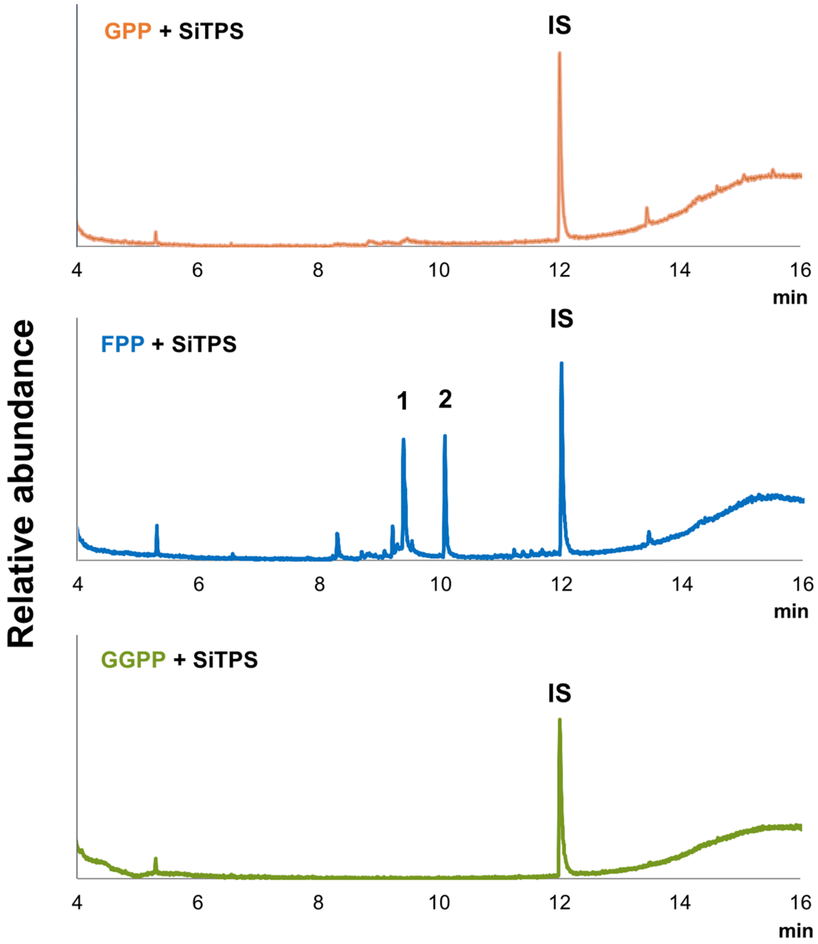

Figure S3. GC-MS chromatograms (total ion count, TIC) from hexane extracts of the *in vitro* enzyme assays of SiTPS with GPP, FPP (*E*,*E*-FPP) and GGPP (*E*,*E*,*E*-GPP) (IS, Internal Standard). GC-MS analysis was done on an Agilent 7890A GC with an Agilent VF-5ms column (30 m × 250 µm × 0.25 µm, with 10m EZ-Guard) and an Agilent 5975C detector. The inlet was set to 280°C splitless injection of 1 µl, helium carrier gas with column flow of 13,8 ml/min. The detector was activated after a four-minute solvent delay. The oven temperature ramp started at 35°C hold for 1 min, increase 20°C/min to 300°C

and hold for 3 min. Obtained spectra from the assay with *E,E*-FPP were compared with NIST17 Mass Spectral Database, suggesting peak 1 is viridiflorene and peak 2 viridiflorol.

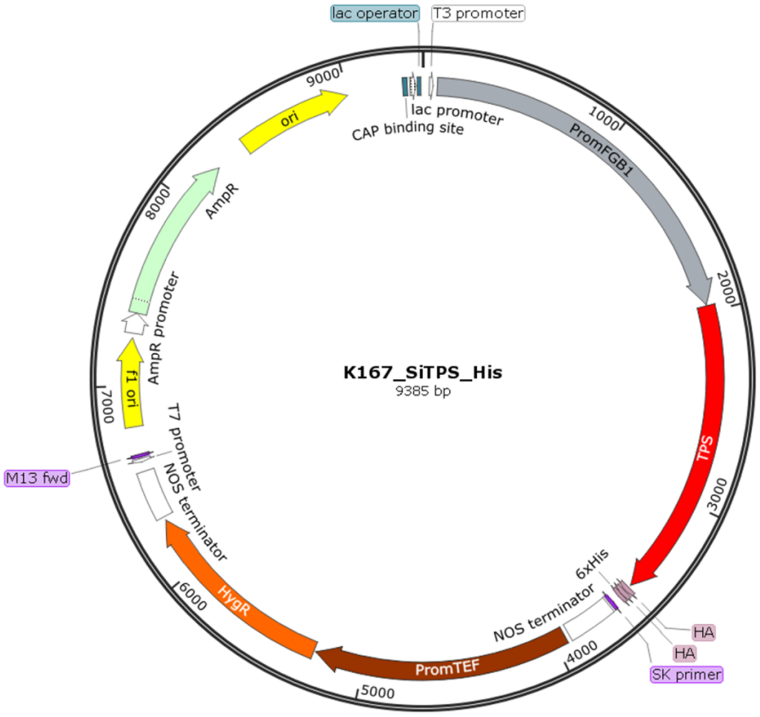

Figure S4. The plasmid K167_PromFCGB1_SiTPS _His [S. Wawra and H. Widmer, unpublished; modified from [46]] used for making the *SiTPS*-overexpressing mutants. The vector backbone is comprised of a hygromycin marker cassette and an ampicillin-resistance gene. *SiTPS* sequence (shown in red) is HA- and His-tagged. Gene expression is under the control of the promoter FCGB1 of S. *indica*.

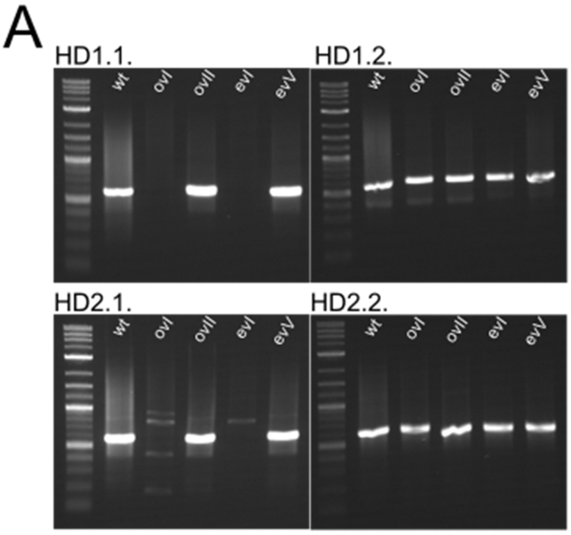

B

S. indica strains	Relative expression ($2^{-\Delta Ct}$±SEM)
**wt**	0.008±0.001[a]
**evV**	0.007±0.001[a]
**ovII**	0.389±0.112[b]

Figure S5. *S. indica* transformants used in colonisation studies were selected based on: **A.** their mating type; gel electrophoresis showing products resulting from the amplification of the four mating type loci HD1.1, HD1.2, HD2.1, HD2.2 performed on gDNA templates extracted from the regenerated transformants. Only heterokaryotic transformants, with all four mating type genes, were used. **B.** *SiTPS* relative expression in transformants grown on CM agar plates for 14 days (wt, wild type; evV, empty vector strain-transformant V; ovII, *SiTPS*-overexpressing strain-transformant II). Relative expression was normalised using *SiGAPDH* (GenBank: FJ810523.1) expression levels and calculated with the $2^{-\Delta Ct}$ method [1,2]. Standard error of the mean (SEM) is also presented (n = 4) and different letters indicate statistical differences (one-way ANOVA in R-studio) at $P \leq 0.05$.

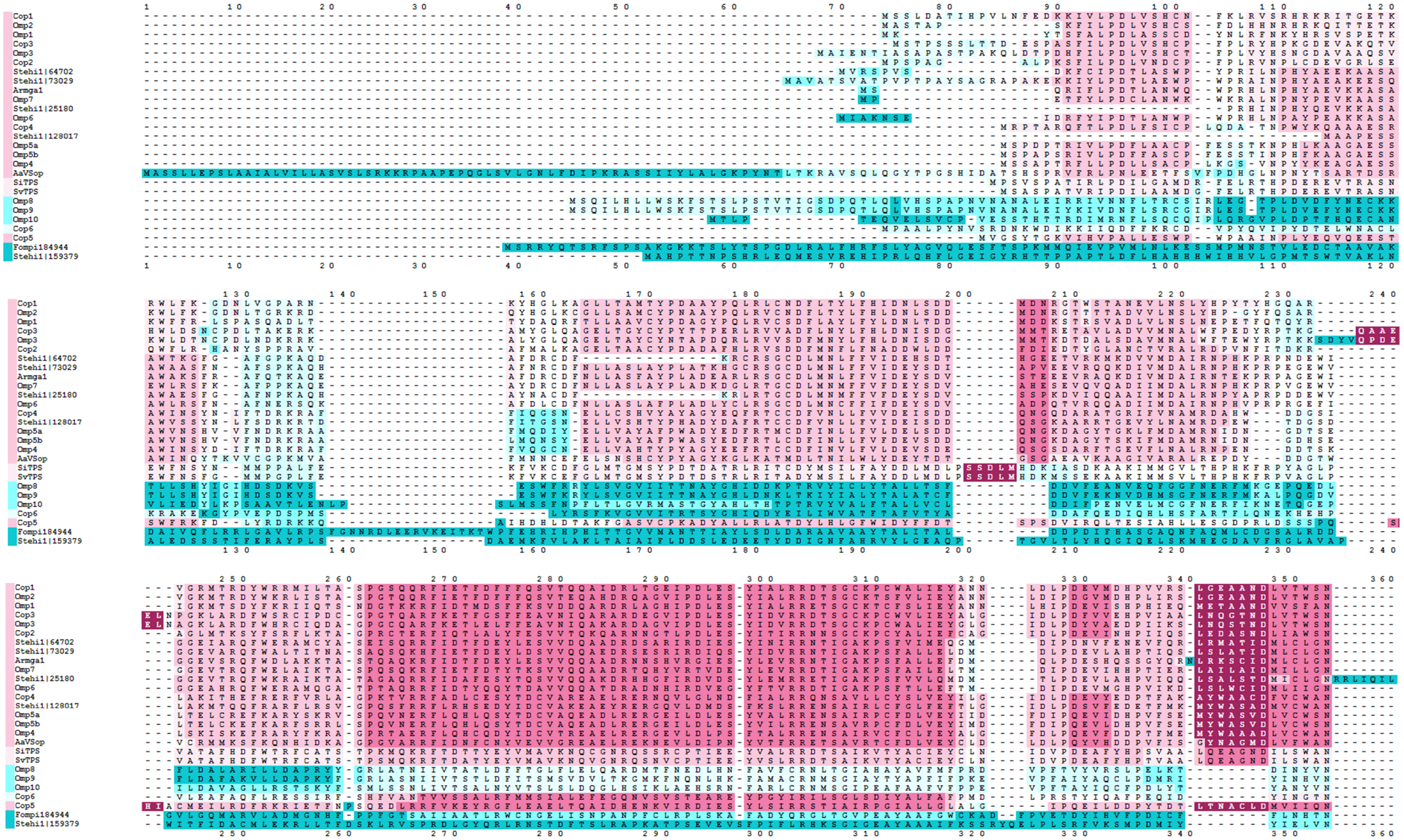
Cop1
Omp2
Omp1
Cop3
Omp3
Cop2
Stehi1|64702
Stehi1|73029
Armga1
Omp7
Stehi1|25180
Omp6
Cop4
Stehi1|128017
Omp5a
Omp5b
Omp4
AaVSop
SiTPS
SvTPS
Omp8
Omp9
Omp10
Cop6
Cop5
Fompi184944
Stehi1|159379

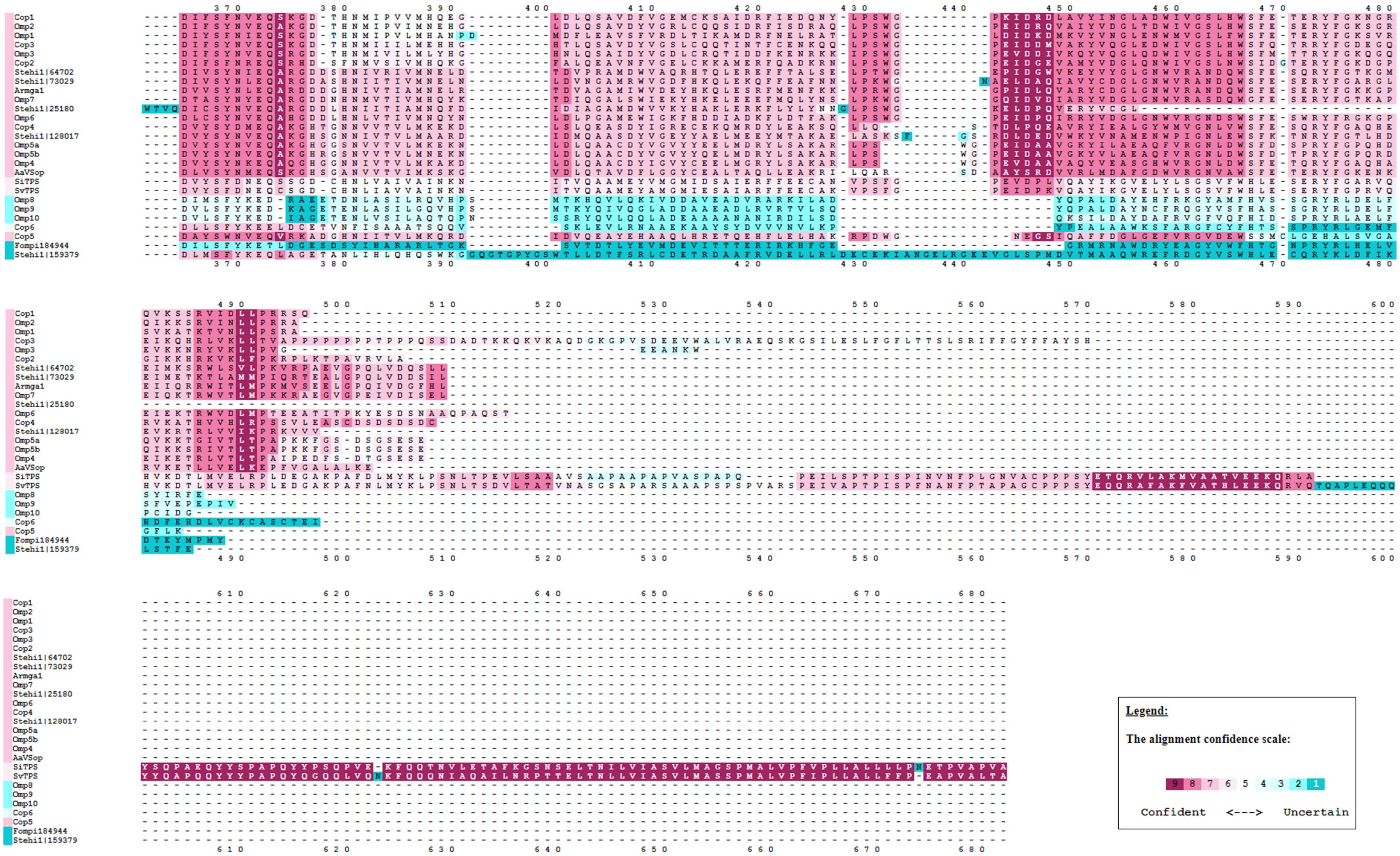
Legend:
The alignment confidence scale:
9 8 7 6 5 4 3 2 1
Confident <---> Uncertain

Figure S6. Multiple Sequence Alignment (MSA) of Basidiomycota STSs, coloured according to the confidence score generated from GUIDANCE2 Server (CLUSTALW). This is a projection of the confidence scores of each residue onto the MSA, using a colour-scale. Shades of magenta indicate confidently aligned residues while shades of blue indicate uncertainly aligned residues (see legend).

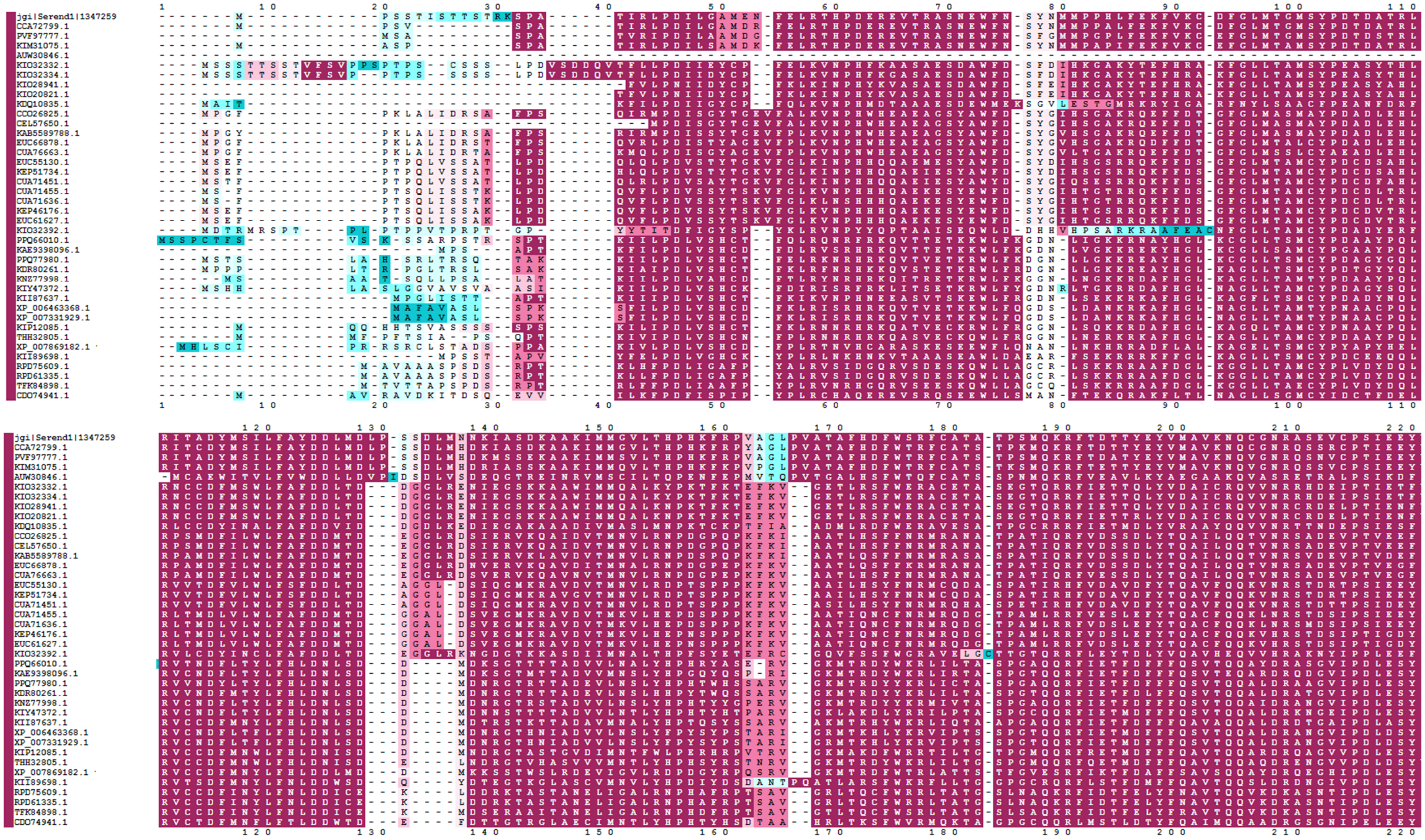
jgi|Serend1|1347259
CCA72799.1
PVF97777.1
KIM31075.1
AUW30846.1
KIO32332.1
KIO32334.1
KIO28941.1
KIO20821.1
KDQ10835.1
CCO26825.1
CEL57650.1
KAB5589788.1
EUC66878.1
CUA76663.1
EUC55130.1
KEP51734.1
CUA71451.1
CUA71455.1
CUA71636.1
KEP46176.1
EUC61627.1
KIO32392.1
PPQ66010.1
KAE9398096.1
PPQ77980.1
KDR80261.1
KNZ77998.1
KIY47372.1
KII87637.1
XP_006463368.1
XP_007331929.1
KIP12085.1
THH32805.1
XP_007869182.1
KII89698.1
RPD75609.1
RPD61335.1
TFK84898.1
CDO74941.1

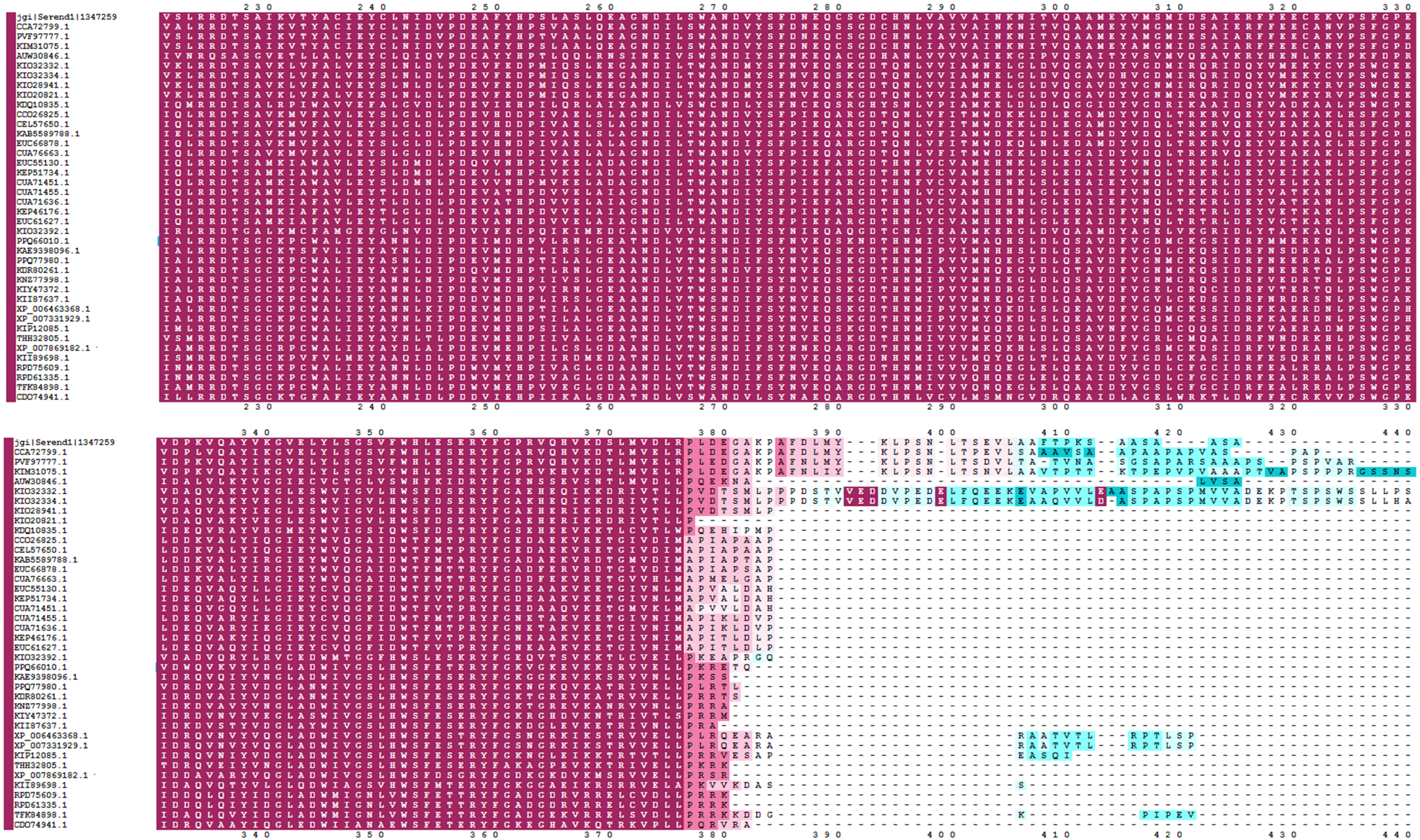
jgi|Serend1|1347259
CCA72799.1
PVF97777.1
KIM31075.1
AUW30846.1
KIO32332.1
KIO32334.1
KIO28941.1
KIO20821.1
KDQ10835.1
CCO26825.1
CEL57650.1
KAB5589788.1
EUC66878.1
CUA76663.1
EUC55130.1
KEP51734.1
CUA71451.1
CUA71455.1
CUA71636.1
KEP46176.1
EUC61627.1
KIO32392.1
PPQ66010.1
KAE9398096.1
PPQ77980.1
KDR80261.1
KNZ77998.1
KIY47372.1
KII87637.1
XP_006463368.1
XP_007331929.1
KIP12085.1
THH32805.1
XP_007869182.1
KII89698.1
RPD75609.1
RPD61335.1
TFK84898.1
CDO74941.1

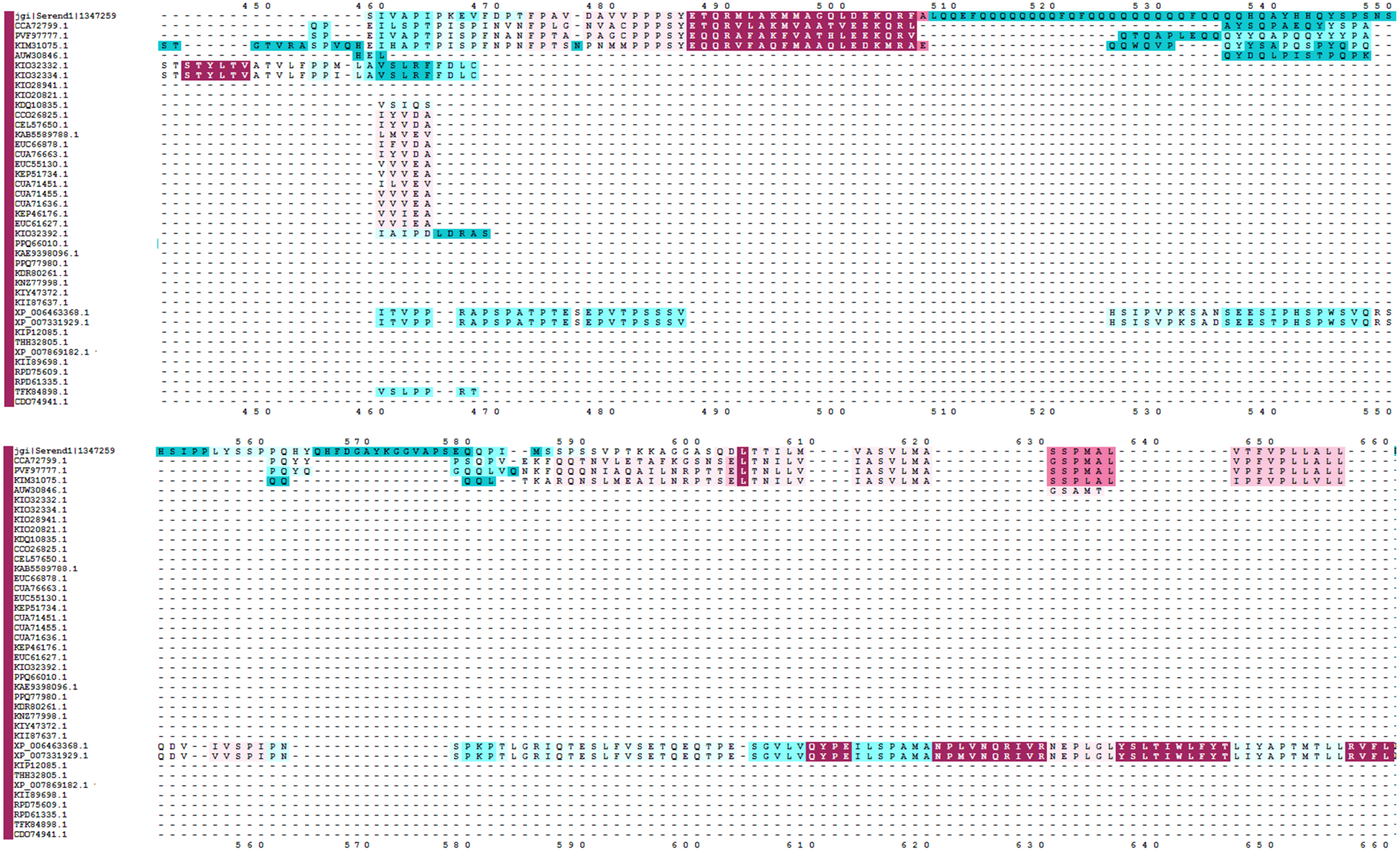
jgi|Serend1|1347259
CCA72799.1
PVF97777.1
KIM31075.1
AUW30846.1
KIO32332.1
KIO32334.1
KIO28941.1
KIO20821.1
KDQ10835.1
CCO26825.1
CEL57650.1
KAB5589788.1
EUC66878.1
CUA76663.1
EUC55130.1
KEP51734.1
CUA71451.1
CUA71455.1
CUA71636.1
KEP46176.1
EUC61627.1
KIO32392.1
PPQ66010.1
KAE9398096.1
PPQ77980.1
KDR80261.1
KNZ77998.1
KIY47372.1
KII87637.1
XP_006463368.1
XP_007331929.1
KIP12085.1
THH32805.1
XP_007869182.1
KII89698.1
RPD75609.1
RPD61335.1
TFK84898.1
CDO74941.1

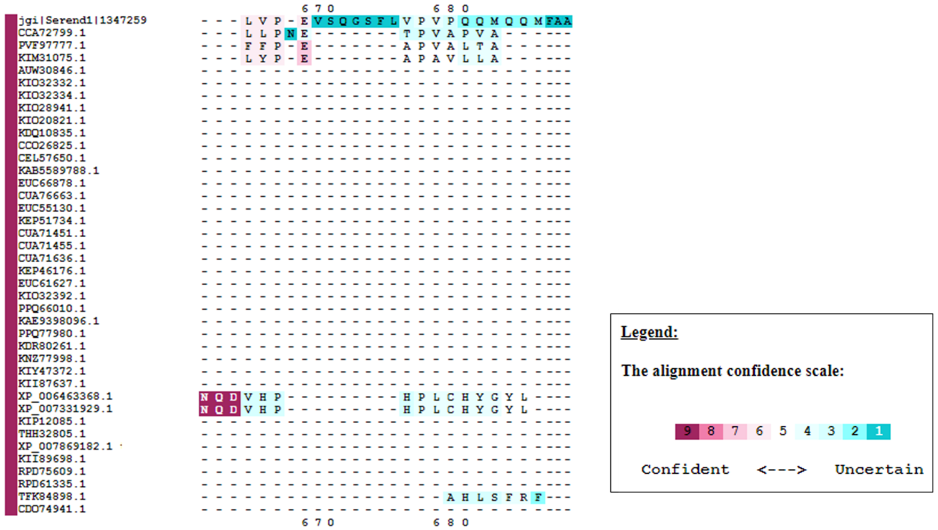

Figure S7. Multiple Sequence Alignment (MSA) of similar to SiTPS proteins, coloured according to the confidence score generated from GUIDANCE2 Server (MAFFT). This is a projection of the confidence scores of each residue onto the MSA, using a colour-scale. Shades of magenta indicate confidently aligned residues while shades of blue indicate uncertainly aligned residues (legend).

Table S1. Primer list

No	Name	Sequence 5′-3′	Used for	Primer efficiency (%)
1	SiTPSinf-F	AGAAGGAGATATACCATGCCA TCTGTCTCTCCTGCCAC	In-fusion® cloning pET_SiTPS	
2	SiTPSinf-R	GGTGGTGGTGCTCGAACGCAAC GGGAGCCACTGGG	In-fusion® cloning pET_SiTPS	
3	TPSovinf-F	CTCCAAAAACAGTCGATGCCAT CTGTCTCTCCTGC	In-fusion® cloning K167_ SiTPS _His	
4	TPSovinfHA -R	TAGATATCGTAGTTTGCAACGG GAGCCACTGGGGT	In-fusion® cloning K167_SiTPS _His	
5	HD1.1-F	CGATACCTACCCGCCTACAA	*S. indica* mating type locus HD 1.1 (PIIN_09915)	
6	HD1.1-R	CTTTTTAAGCGGTGCTGGAG	*S. indica* mating type locus HD 1.1 (PIIN_09915)	
7	HD2.1-R	ATGAGTACGATTGCCCAAGG	*S. indica* mating type locus HD 2.1 (PIIN_09916)	
8	HD2.1-F	TCGTCTCGTAGGCGACTTTT	*S. indica* mating type locus HD 2.1 (PIIN_09916)	
9	HD1.2-R	AGATATCCGGAGGCGAGTTT	*S. indica* mating type locus HD 1.2 (PIIN_09977)	
10	HD1.2-F	CCTGAATCTGCTGTTCGTCA	*S. indica* mating type locus HD 1.2 (PIIN_09977)	
11	HD2.2-F	ACATCTGGCTCCCATTTACG	*S. indica* mating type locus HD 2.2 (PIIN_09978)	
12	HD2.2-R	GTTGAGCTTTGGCTCGTTTC	*S. indica* mating type locus HD 2.2 (PIIN_09978)	
13	SiGAPDH-F	TCGGTCGTATTGGCCGTATC	*SiTPS* relative expression- Normalization	
14	SiGAPDH-R	CTCGATAACATAGTCGACACCT	*SiTPS* relative expression- Normalization	110
15	SiTPS-F	CAACAGCTACAACATGATGC	*SiTPS* relative expression	
16	SiTPS-R	GTCCAGAAGTCATGGAAAGC	*SiTPS* relative expression	100
13	SiITS-F	CAACACATGTGCACGTCGAT	*S. indica* ITS estimation of fungal biomass	
14	SiITS-R	CCAATGTGCATTCAGAACGA	*S. indica* ITS estimation of fungal biomass	
15	SlTUB-F	AACCTCCATTCAGGAGATGTTT	*Solanum lycopersicum* β-tubulin estimation of plant biomass	
16	SlTUB-R	TGCTGTAGCATCCTGGTATT	*Solanum lycopersicum* β-tubulin estimation of plant biomass	
